# Editorial: The impact of the COVID-19 pandemic on dermatology patients: diagnosis, treatment, and prognosis

**DOI:** 10.3389/fmed.2024.1418722

**Published:** 2024-05-02

**Authors:** Huining Huang, Ziyu Guo, Danyao Chen, Guangtong Deng

**Affiliations:** ^1^Department of Dermatology, Xiangya Hospital, Central South University, Changsha, China; ^2^National Engineering Research Center of Personalized Diagnostic and Therapeutic Technology, Changsha, Hunan, China; ^3^Furong Laboratory, Changsha, Hunan, China; ^4^Hunan Key Laboratory of Skin Cancer and Psoriasis, Hunan Engineering Research Center of Skin Health and Disease, Xiangya Hospital, Central South University, Changsha, China; ^5^National Clinical Research Center for Geriatric Disorders, Xiangya Hospital, Changsha, Hunan, China; ^6^Department of Thoracic Surgery, Xiangya Hospital, Central South University, Changsha, Hunan, China

**Keywords:** COVID-19, dermatology, diagnosis, treatment, prognosis

The coronavirus disease 2019 (COVID-19) pandemic has posed a profound impact on the global healthcare systems, including the field of dermatology (1, 2). The causative virus, SARS-CoV-2, primarily targets the respiratory system and compromises the immune system, which can trigger immune-related skin disorders or aggravate pre-existing skin conditions (3–5). Concurrently, the pandemic has reshaped medical practice and patient behaviors worldwide, leading to a notable reduction in dermatology admissions and extended hospital stays for severe cases due to concerns about hospitalization and associated treatment (6, 7). Moreover, vaccination against COVID-19 have been associated with dermatological manifestations, including dermatomyositis and new or recurrent immune-related skin diseases (8–11). This editorial introduces a Research Topic of seven papers that delve into the impact of the COVID-19 pandemic and COVID-19 vaccines on the diagnosis, treatment, and outcomes of dermatological conditions.

In a comprehensive retrospective study, Kalanj et al. analyzed the total number of hospitalized patients with skin diseases, as well as those who underwent conservative treatment and surgical interventions, comparing periods before and during the COVID-19 pandemic. Their findings highlight a significant reduction in hospitalizations and surgical procedures (with the exception of breast reconstruction) during the pandemic. This reduction is largely attributable to the state-enacted pandemic prevention and control measures, including social distancing, travel restrictions, and partial or complete lockdowns. Apostu et al. conducted a retrospective cohort study focusing on the number of diagnosed melanoma patients before and after the pandemic, as well as the age, gender, histological characteristics of confirmed cases. They observed a substantial decline in the incidence of new melanoma cases following the COVID-19 pandemic. Additionally, the study found that patients diagnosed with melanoma during the pandemic were older and exhibited more severe prognostic features, such as higher Breslow indexes, increased mitotic counts, and greater ulceration and thickness. These findings suggest that the pandemic has not deterred patients with more aggressive forms of melanoma from seeking treatment, despite the overall decrease in healthcare engagement.

Huang et al. collected and analyzed data on psoriasis patients receiving targeted biologic maintenance therapy during the COVID-19 pandemic, focusing on the comparative efficacy of different biological agents. They pointed out that biological agents targeting the IL-17 and IL-23 pathways are more effective than those targeting TNF-α (Huang et al.). This difference in efficacy may be due to pathogenic roles of IL-23 and IL-17 in both COVID-19 and psoriasis. Furthermore, an exacerbation of psoriasis could heighten the IL-17-mediated immune response, potentially increasing the severity of COVID-19 in these patients. This study provides crucial guidance for selecting targeted therapeutic drugs for psoriasis patients, particularly those with concurrent COVID-19 infection, during the pandemic.

COVID-19 vaccines can also lead to the new occurrences or recurrences of immune-related skin diseases. Cowan et al. identified a potential increase in the risk of new occurrences or recurrences of autoimmune bullous diseases (AIBDs) following COVID-19 vaccination. This discovery serves as a reminder for dermatology clinicians to inquire about patients' recent vaccination history when treating patients with AIBDs. Similarly, Ghanaapisheh et al. noted a possible association between COVID-19 vaccinations, especially mRNA vaccines, and the occurrence of bullous pemphigoid (BP). Notably, the majority of BP patients remain unaffected by COVID-19 vaccinations and even those experiencing worsening conditions typically do not face severe side effects, highlighting the evidence-based safety of vaccines. Olszewska et al.'s reviewed the potential link between COVID-19 vaccination and primary cutaneous lymphoma (CL). Their analysis of data from 24 patients across various studies indicates that primary cutaneous CD30-positive lymphoproliferative disorders are the most prevalent type of CL following COVID-19 vaccination. Ghanaapisheh et al. also highlighted the potential risk of mRNA vaccine induced-CL. Therefore, researchers specifically advise patients with a history of lymphoproliferative diseases to monitor their health closely post-COVID-19 vaccination and to remain vigilant for any signs of disease progression.

Kartal et al. explored the effects of COVID-19 vaccine on patients with chronic spontaneous urticaria (CSU), observing a significant increase in the median Urticaria Activity Score post-vaccination compared to pre-vaccination levels. Their study also documented cases where individuals developed vascular edema and allergic reactions subsequent to receiving the vaccine. These findings emphasize the potential side effects associated with COVID-19 vaccines. Dermatologists are therefore urged to remain vigilant and consider the possibility of new or recurring immune-related skin conditions in patients who have been vaccinated against COVID-19.

In summary, this Research Topic outlines the multifaceted effects of the COVID-19 pandemic on the occurrence, development, diagnosis, and treatment of various skin diseases. Firstly, there has been a notable decline in the total number of hospitalized patients with skin diseases and in surgical patients, which provides valuable data for hospitals looking to optimize their service system structure. Secondly, there appears to be an increase in the aggressiveness of melanoma during the pandemic, likely due to delays in diagnosis and treatment. Thirdly, the use of biological agents targeting IL-17 and IL-23 has proven more effective than those targeting TNF-α during pandemic, for reasons yet to be determined. Finally, there is a suggested link between COVID-19 vaccination and the onset of autoimmune bullous diseases, chronic spontaneous urticaria, or primary skin lymphoma. Exploring the potential mechanisms behind these associations could enhance our understanding of the development and progression of these conditions.

## Author contributions

HH: Writing – original draft, Writing – review & editing. ZG: Writing – original draft, Writing – review & editing. DC: Writing – original draft, Writing – review & editing. GD: Writing – original draft, Writing – review & editing.
